# Bibliographical Department

**Published:** 1880-09

**Authors:** 


### BIBLIOGRAPHICAL DEPARTMENT.

"Nothing extenuate, nor set down aught in malice."

A Treatise on Oral Deformities as a Branch of Mechanical Surgery; by Norman W. Kingsley, M. D. S., D. D. S., President of the Board of Censors of the State of New York; late dean of the New York College of Dentistry and Professor of Dental Art and Mechanism; Member of the American Academy of Dental Science, of the Odontological Society of Pennsylvania and of the Odontological Society of New York, etc., with over 250 illustrations: New York: D. Appleton & Company, 1, 3 & 5 Bond street; 1880. Price $5.00.

We are in receipt of the above work, and, like everything else emanating from the Messrs. Appleton's house, it is neatly and artistically gotten up. With its copious index, the book is well suited for easy and direct reference to any of the subjects it contains, and the cuts and other illustrations, render the text clearly intelligible. It is written in a flowing, practical style and in language so plain as to make what the author says, easy of comprehension by any one. It supplies a want in this department of dentistry, that has been felt for a long while, and we have no hesitation in commending it to every practitioner who desires to keep fully abreast with the times in which we live.

This work demonstrates conclusively, that dentistry can no longer be considered a merely mechanical art, or trade, but a *Surgical Specialty* of the highest value and importance to the civilization of the present age of the world. Having made oral deformities a specialty, we feel more competent to appreciate Dr. Kingsley's great work. We heartily commend the book to our readers. B. M. W.

"A New School Physiology. By Richard J. Dunglison, M. D., author of "the Practitioner's Reference Book;" editor of "Dunglison's Medical Dictionary," "History of Medicine," Secretary of the American Academy of Medicine," etc., etc., illustrated with one hundred and seventeen engravings. Published by Porter & Coates, Philadelphia." Is the title page of a very neat and handsomely printed volume of 314 pages. Considerable experience as lecturer on Physiology in the Grammar Schools of this city, and acquaintance with the books prepared heretofore for popular use in our schools, enable us to speak with confidence in regard to Dr. Dunglison's book. The arrangement, the cuts, the questions on the text, and the style, are all admirably well suited for the purpose the book was intended. As such we heartily commend it to all those engaged in teaching the young who are sufficiently advanced to receive intelligent ideas of the structure and functions of their bodies, as in every way worthy the important subject on which it treats.

"A Practical Treatise on Tumors of the Mammary Gland, *Embracing their History Pathology, Diagnosis and Treatment.* By Samuel W. Gross, A. M., M. D., Surgeon to, and Lecturer on Clinical Surgery in the Jefferson Medical College Hospital, and the Philadelphia Hospital; President of the Pathological Society of Philadelphia; Fellow of, and formerly Mutter Lecturer on Surgical

Pathology in the College of Physicians of Philadelphia; Fellow of the Academy of Surgery in Philadelphia, etc., illustrated by seventy-nine engravings. New York: D. Appleton and Company, 1, 3 and 5 Bond street, 1880." The foregoing is the title of a new book just issued by the enterprising and widely famed publishing house of the Messrs. Appleton, and it is hardly necessary to add, their work has been admirably performed. The author gracefully and properly dedicates the volume to his distinguished father, Professor S. D. Gross, and has given us one of the best and most complete treatises on the subject in our vernacular literature! As such we heartily endorse the book to the profession, containing as it does all that is valuable and necessary to be known upon the subject at the present day.

THE NORTH AMERICAN REVIEW, Edited by Allen Thorndike Rice, and published by the Messrs. Appleton, for August and September, has been received and gladly placed upon our exchange list. This old and standard Review is too well known to require any advertisement or praise from us; but we cannot allow this opportunity to pass without calling attention to the fact that the August issue contains an introductory chapter on the "Ruined Cities of Central America." The September nnmber introduces the first article on that absorbingly interesting subject by M. Charnay, the chief of the grand expedition now engaged in the investigation and study of those wonderful remains of an unknown and highly intelligent people. These papers alone will be worth many times the subscription price of the "Review."

PAMPHLETS, &c.—Several pamphlets and two or three new Journals were received before this department was ready for the printers; but in the confusion incident to the removal of our professional office and residence, a few days since, from 147 Edmonson avenue to 225 N. Gilmor street, they became misplaced. Will our friends be kind enough to send us duplicate copies of them? H. L. B.

THE MEDICAL AND SURGICAL DIRECTORY of the State of Iowa, for 1880–81.

We beg to acknowledge our indebtedness to the author, Dr. Charles H. Lothrop, of Lyons, Iowa, for a copy of the above book. It supplies a large amount of information that might be utilized to the benefit of the profession, beyond the territorial boundaries of the State of Iowa.

Through it, all practitioners of medicine in that State may become acquainted with the address and professional status of one another, and the "fee tables" governing practice in the commonwealth. All the counties in the State are arranged alphabetically, and the practitioners in each county, whether "regular, irregular or defective," are disposed of in like manner, with the date and school from which they received their degree, when they are graduates. While the "regulars" largely preponderate, we were still surprised to find so many of the "irregular" and "defective" sort engaged in practice in that State! It is a little strange that some of the latter class should have been found without a "diploma" when Buchanan, and Payne, were in their glory, and supplying the magical "sheepskin" at moderate rates, whether the applicant ever saw the interior of a medical school or not! We would like to see an "*exposition*" of the credentials of the practitioners of the Monumental City, put forth in a similar manner? The book contains much other valuable information, more particularly interesting however, to medical men of that state.

We are also in the receipt of a pamphlet on MALARIA, from the same author, which we have read with interest. The following have also been received, viz: First Decemmial Report of the "Manhattan Eye and Ear Hospital, New York City."

"A New Eye and Ear Bandage. By Samuel Theobald, M. D., of Baltimore, Md."

"Minutes of the 24th and 25th Annual Meetings of the State Medical Society of Kentucky, 1878 and 1879."

"Sixtieth Annual Catalogue and Announcement of the Medical College of Ohio."

"Sixth Anniversary Announcement of the Medical Department of Tennessee, Nashville Medical College."

"Gastro-Hystereotomy, or the Recent Modication of the Cæsarean Section, by Dr. Perro. By Isaac E. Taylor, M. D., Emeritus Professor of Obstetrics, and of the Diseases of Women and Children, Bellevue Hospital Medical College, New York."

We are indebted to Dr. J. A. McArthur, Fellow of the Massachusetts Medical Society, etc., for a copy of his monograph, entitled "Consumption and Tuberculoses, Notes on their Treatment by the Hypophosphites." Second Edition.

"Peptonised Milk as Food for Infants and Invalids. By R. J. Nunn, M. D., Prof. Theory and Practice of Medicine in the Savannah Medical College, Ga."

This Re-print from the *American Journal of Obstetrics*, etc., will well repay perusal, as it contains suggestions of value to every one. We will have the pleasure of presenting an original article on Diphtheria from our old friend, the author, in the present issue of the Journal, and hope he may find time to render similar service very often in the future.

"National Sanitation. By J. C. LeHardy, M. D., Savannah." Reprint from the Transactions of the Medical Association of Georgia. We regret that accident prevented this able and highly valuable paper of Prof. LeHardy from appearing first in the pages of this Journal. We hope our friend and whilom confrere, may be able to include in another paper, statistics he could not obtain for this, and permit our Journal to become the medium of placing or laying it before the profession? We know of no one better qualified than he is, or who would be more conscientious in the performance of so important a work.

"The Sanitarian. A Monthly Magazine, devoted to the Preservation of Health, Mental and Physical Culture, edited by Drs. A. N. Bell and T. P. Corbally. Published by A. N. Bell, A. M., M. D., No. 8 Prince street. New York, 3 dollars per annum." This old and well established Journal is fully up to the times on all it undertakes to treat, and is well and ably edited.

"The Australian Medical Journal, May 15th, 1880, published at Melbourne, by Stillwell & Co., 78 Collins street, east, on the 15th of each month, Subscription 1£ per annum." The number before us is well filled with interesting articles, original and selected, and we beg leave to say to our antipodal confrere that we take pleasure in adding *The Australian Medical Journal* to our list of exchanges.

ANALES DE LA SOCIEDAD ODONTOLOGICA.—De La Habana. Revista Meresula, comite Redactor Para, 1880-81, Drs. Cirilo A. Yarini,—Pedro Calvo—Francisco N. Justiniani—Manual A. Agnilera. Florencio J. Cancio. Ano 11 Junio De 1880.—Num. 3 Imprenta "La Prueba," Amargura 77. Habana.

NEBRASKA STATE DENTAL SOCIETY will hold its fourth annual meeting at Omaha, on Tuesday, September 21st, at 7.30 P. M.

W. F. ROSEMAN, *Rec. Sec't'y.*

THE DENTAL ASSOCIATION OF THE UNITED STATES OF AMERICA, was organized the second week of August, 1880, in the City of New York. A. L. Northrop, of N. Y., President; R. Finley Hunt, of Washington, Secretary; H. B. Noble, of Washington, Treasurer. The projectors of this association present some good and new features, which we hope will result in obtaining the aid of the U. S. Government in securing such information and material relating to the specialty of dentistry as will advance the science, as well as give Dental Surgeons appointments in the army and navy. It is claimed to be purely national, and its library, museum, &c., are to be in a suitable building in the City of Washington.

---

ANNUAL ANNOUNCEMENT OF COLLEGES RECEIVED, SESSION 1880-81:

Philadelphia Dental College; also Hospital of Oral Surgery, James E. Garrison, D. D. S., M. D., Dean, Philadelphia, Pa.

New York College of Dental Surgery, Frank Abbott, M. D., Dean, N. Y. City.

Ohio College of Dental Surgery, H. A. Smith, D. D. S., Dean, Cincinnati.

Dental College of the University of Michigan, J. Taft, A. M., D. D. S., Dean, Ann Arbor.

Royal College of Dental Surgeons. of Ontario, L. Teskey, M. B., Registar of Faculty.

